# The parietal architecture binding cognition to sensorimotor integration: a multimodal causal study

**DOI:** 10.1093/brain/awad316

**Published:** 2023-09-16

**Authors:** Luca Fornia, Antonella Leonetti, Guglielmo Puglisi, Marco Rossi, Luca Viganò, Bianca Della Santa, Luciano Simone, Lorenzo Bello, Gabriella Cerri

**Affiliations:** Department of Medical Biotechnology and Translational Medicine, MoCA Laboratory, Università degli Studi di Milano, Milano, 20122, Italy; Department of Oncology and Hemato-Oncology, Neurosurgical Oncology Unit, Università degli Studi di Milano, Milano, 20122, Italy; Department of Medical Biotechnology and Translational Medicine, MoCA Laboratory, Università degli Studi di Milano, Milano, 20122, Italy; Department of Medical Biotechnology and Translational Medicine, MoCA Laboratory, Università degli Studi di Milano, Milano, 20122, Italy; Department of Oncology and Hemato-Oncology, Neurosurgical Oncology Unit, Università degli Studi di Milano, Milano, 20122, Italy; Department of Medical Biotechnology and Translational Medicine, MoCA Laboratory, Università degli Studi di Milano, Milano, 20122, Italy; Department of Medicine and Surgery, Università Degli Studi di Parma, Parma, 43125, Italy; Department of Oncology and Hemato-Oncology, Neurosurgical Oncology Unit, Università degli Studi di Milano, Milano, 20122, Italy; Department of Medical Biotechnology and Translational Medicine, MoCA Laboratory, Università degli Studi di Milano, Milano, 20122, Italy

**Keywords:** apraxia, motor cognition, sensorimotor integration, dorsal stream

## Abstract

Despite human’s praxis abilities are unique among primates, comparative observations suggest that these cognitive motor skills could have emerged from exploitation and adaptation of phylogenetically older building blocks, namely the parieto-frontal networks subserving prehension and manipulation. Within this framework, investigating to which extent praxis and prehension-manipulation overlap and diverge within parieto-frontal circuits could help in understanding how human cognition shapes hand actions. This issue has never been investigated by combining lesion mapping and direct electrophysiological approaches in neurosurgical patients.

To this purpose, 79 right-handed left-brain tumour patient candidates for awake neurosurgery were selected based on inclusion criteria. First, a lesion mapping was performed in the early postoperative phase to localize the regions associated with an impairment in praxis (imitation of meaningless and meaningful intransitive gestures) and visuo-guided prehension (reaching-to-grasping) abilities. Then, lesion results were anatomically matched with intraoperatively identified cortical and white matter regions, whose direct electrical stimulation impaired the Hand Manipulation Task.

The lesion mapping analysis showed that prehension and praxis impairments occurring in the early postoperative phase were associated with specific parietal sectors. Dorso-mesial parietal resections, including the superior parietal lobe and precuneus, affected prehension performance, while resections involving rostral intraparietal and inferior parietal areas affected praxis abilities (covariate clusters, 5000 permutations, cluster-level family-wise error correction *P* < 0.05). The dorsal bank of the rostral intraparietal sulcus was associated with both prehension and praxis (overlap of non-covariate clusters). Within praxis results, while resection involving inferior parietal areas affected mainly the imitation of meaningful gestures, resection involving intraparietal areas affected both meaningless and meaningful gesture imitation. In parallel, the intraoperative electrical stimulation of the rostral intraparietal and the adjacent inferior parietal lobe with their surrounding white matter during the hand manipulation task evoked different motor impairments, i.e. the arrest and clumsy patterns, respectively.

When integrating lesion mapping and intraoperative stimulation results, it emerges that imitation of praxis gestures first depends on the integrity of parietal areas within the dorso-ventral stream. Among these areas, the rostral intraparietal and the inferior parietal area play distinct roles in praxis and sensorimotor process controlling manipulation. Due to its visuo-motor ‘attitude’, the rostral intraparietal sulcus, putative human homologue of monkey anterior intraparietal, might enable the visuo-motor conversion of the observed gesture (direct pathway). Moreover, its functional interaction with the adjacent, phylogenetic more recent, inferior parietal areas might contribute to integrate the semantic-conceptual knowledge (indirect pathway) within the sensorimotor workflow, contributing to the cognitive upgrade of hand actions.

## Teaser

By combining lesion mapping and direct electrophysiological approaches in neurosurgical patients, Fornia *et al.* show that the integrity of specific parietal areas is crucial for binding cognition into the sensorimotor workflow. The findings may have implications for the treatment of brain injuries and neurodegenerative diseases.

## Introduction

Praxis is the neurological process by which cognition directs motor action.^1,2^ This process allows an abstract, conceptual and extremely flexible use of our hand sensorimotor repertoire, which is considered a hallmark of the human evolution. Praxis embraces several skills, from the ability to functionally interact with tools or pantomime their use (transitive actions), to imitation of meaningless and meaningful gestures (intransitive actions). These skills can be dramatically impaired following brain lesions resulting in the so-called ‘apraxia’, a deficit in the execution of purposive hand movements, not attributable to elementary motor and sensory disorders.

Converging comparative evidence suggests that phylogenetic old brain mechanisms subserving transitive actions, such as object prehension and manipulation, may represent the building blocks from which the human praxis abilities have emerged.^3-5^ Specifically, in this regard, humans and non-human primates share similar dorso-dorsal and dorso-ventral parieto-frontal streams for controlling distinct, although complementary, aspects of the hand-object oriented actions.^6,7^ In humans, the presence of the building blocks and the parallel expansion of the frontal, parietal and temporal areas^8^ led to a significant sophistication of the sensorimotor repertoire, which represents the substrate fostering in humans a rapid cultural evolutionary process. Coherent with this view, functional MRI (fMRI) studies showed that the hand-related parieto-frontal connectivity extends in humans to compose the so-called praxis representation network (PRN^9,10^). The human PRN is a large-scale, left-lateralized, temporo-parietal-frontal circuit claimed to be involved in translating conceptual and sensorimotor information into purposeful hand skilled acts (praxis), including transitive and intransitive hand gestures.^9,11^ Pivotal lesion studies in stroke patients support the evidence that impairment of specific parietal and temporal sectors of this large-scale pathway results in the onset of distinct apraxia symptoms.^12-15^

Overall, these results suggest that the exploitation and modification of the pre-existing parieto-frontal building blocks under the guidance of evolutionary processes^16-18^ has been critical for the achievement of sophisticated and cognitively directed hand actions. Among them, intransitive ‘communicative’ hand-arm gestures represent a distinguishing feature of humans with respect to the monkeys that hardly use their hand for such purposes.^19^ Within this framework, investigating to what extent the pathways subserving object-oriented actions (including visuo-guided prehension and object-manipulation) and the pathways subserving intransitive praxis gestures overlap and differentiate within the parieto-frontal circuits, seems crucial to disclose the neural mechanisms shaping the motor action based on high-level cognitive information.

To this aim, the present study was grounded in the clinical setup for patients undergoing awake neurosurgery for brain tumour resection allowing the use of complementary causal approaches and specifically (i) lesion symptom mapping (LSM); and (ii) intraoperative direct electrical stimulation (DES). Specifically in this study, the LSM was performed in the early postoperative phase (7 days post-surgery) to localize the brain regions associated with lower scores in praxis (imitation of meaningless and meaningful intransitive hand gestures) and prehension (reaching-to-grasping) performance. The regions highlighted by the LSM were then anatomically matched with cortical and white matter regions related to haptic hand-object manipulation identified intraoperatively with DES within the same cohort of patients. The intraoperative hand manipulation task (HMt) was performed without visual guidance in order to isolate and preserve mainly motor (and/or somatomotor) components of the parieto-premotor areas subserving hand manipulation functions.^20-26^ Primary input to the rationale of this study has been the observation that the application of the intraoperative HMt actually turned out to also reduce long-term upper limb ideomotor apraxia deficits. This observation suggests a close proximity between manipulation and praxis substrates, fostering the hypothesis that the exploitation of specific hand-related building blocks might be an important aspect for the emergence of cognitive praxis gestures as well as a relevant clinical tool guiding the intraoperative monitoring. However, even though permanent apraxia was avoided, ∼20% of patients suffered transient ideomotor apraxia symptoms in the early postoperative phase.^20^ These transient symptoms may be explained by the marginal impairment of the praxis-related neural substrates, possibly interleaved with motor and/or somatomotor object-manipulation substrates and/or lying in the tissue along the resection cavity’s borders draw with the HMt. In particular, in the early postoperative phase, the resection borders undergo a transient inflammation, possibly altering their correct functioning.

Overall, these clinical considerations fostered the need to investigate the degree of co-localization between the intraoperative DES sites associated with the HMt, and the regions associated with lower scores in praxis functions in the early postoperative phase. However, the intraoperative HMt involved only distal control of the hand-object interaction, therefore the full deployment of the visuo-guided prehension (from the direction of arm movements to the shaping of the hand according to the object shape and location), i.e. reaching-to-grasping, was intraoperatively unexplored. To fill this gap and provide the most comprehensive view of all the areas involved in the hand-object oriented action (from proximal-reaching to distal-grasping/manipulation), the spatial matching between the regions associated with lower scores in visuo-guided prehension assessment and the intraoperative hand manipulation-related sectors was performed. Notably, the complementary use of three tasks (HMt, visuo-guided prehension and praxis) all constrained to the dexterous use of the hand as a final common path, grounds on four pillars: (i) the tasks rely on different sensory and sensorimotor modalities: visuomotor (both prehension and praxis) and somatosensory-motor (or somatomotor, all the tasks); (ii) the tasks investigated distinct hand-action domains: transitive hand-object oriented actions (HMt and prehension) and imitation of intransitive gesture (praxis); (iii) within transitive action, the haptic execution of the HMt investigated the somatomotor component involved in the hand-object oriented action, while the visuo-guided prehension extended the investigation to the visuomotor component; and (iv) within praxis, gestures to be imitated differed in the cognitive content: communicative (meaningful) or not (meaningless).

The combined use of the tasks along the four pillars allowed us to investigate the degree of co-localization between the areas involved in transitive object-related actions (HMt and prehension) and the areas more specifically involved in the imitation of the observed intransitive action. The combination of these tasks within the same framework aims to investigate to what extent the intransitive praxis gestures, requiring the purposeful use of the hand for imitating meaningless or meaningful (communicative) gesture, exploit phylogenetically ancient parieto-frontal pathways subserving transitive object-oriented actions.

## Materials and methods

### Patient selection

Enrolled in the study were 79 right-handed patients undergoing awake neurosurgery for a left-brain tumour resection [WHO tumour grade: high-grade glioma (HGG) *n* = 48, 61%; low-grade glioma (LGG) *n* = 27, 34%; others *n* = 4, 5%; age: average = 49.5 ± 14.8, range 19–76 years; gender: male *n* = 55, 69%; female *n* = 24, 31%]. All patients were assessed for handedness using the Edinburgh Handedness Inventory and underwent a preoperative, 7-day and 1-month postoperative neuropsychological evaluation and objective neurological examinations. The 79 patients met the following inclusion criteria: (i) first procedure of tumour resection, to minimize the impact of the disease and treatments on brain functional reorganization; (ii) tumour not infiltrating the supplementary motor area, the precentral/postcentral hand-knob in order avoid the inclusion of patients with invalidating basic motor and somatosensory impairments; (iii) postoperative MRC (Medical Research Council) upper limb score ≥4 to ensure the absence of elementary motor deficits affecting the praxis and prehension assessments; (iv) postoperative absence of severe sensory (tactile and visual) deficit assessed by means of neurological assessment; (v) postoperative absence of language comprehension deficits impacting the reliability of the assessments; (vi) preoperative absence of pathological score for ideomotor apraxia (De Renzi global score >53); and (vii) preoperative absence of any clinically observable deficit during object prehension-manipulation [Action Research Arm Test (ARAT) global score = 48].

According to tumour localization, patients were categorized mainly as frontal/fronto-temporal (*n* = 27, mean cavity volume: 113.378 ± 75.868 voxels, range 3.607–259.376), parietal/parieto-temporal (*n* = 34, mean cavity volume: 48.528 ± 32.429 voxels, range 12.949–152.927) and temporal (*n* = 18, mean cavity volume: 61.679 ± 38.321 voxels, range: 13.952–135.001).

All participants gave written informed consent to the surgical mapping procedure (IRB1299) and data analysis for research purposes, followed the principles outlined in the Declaration of Helsinki.

### Intraoperative mapping with the hand-manipulation task and workflow of the study

**Figure 1:**
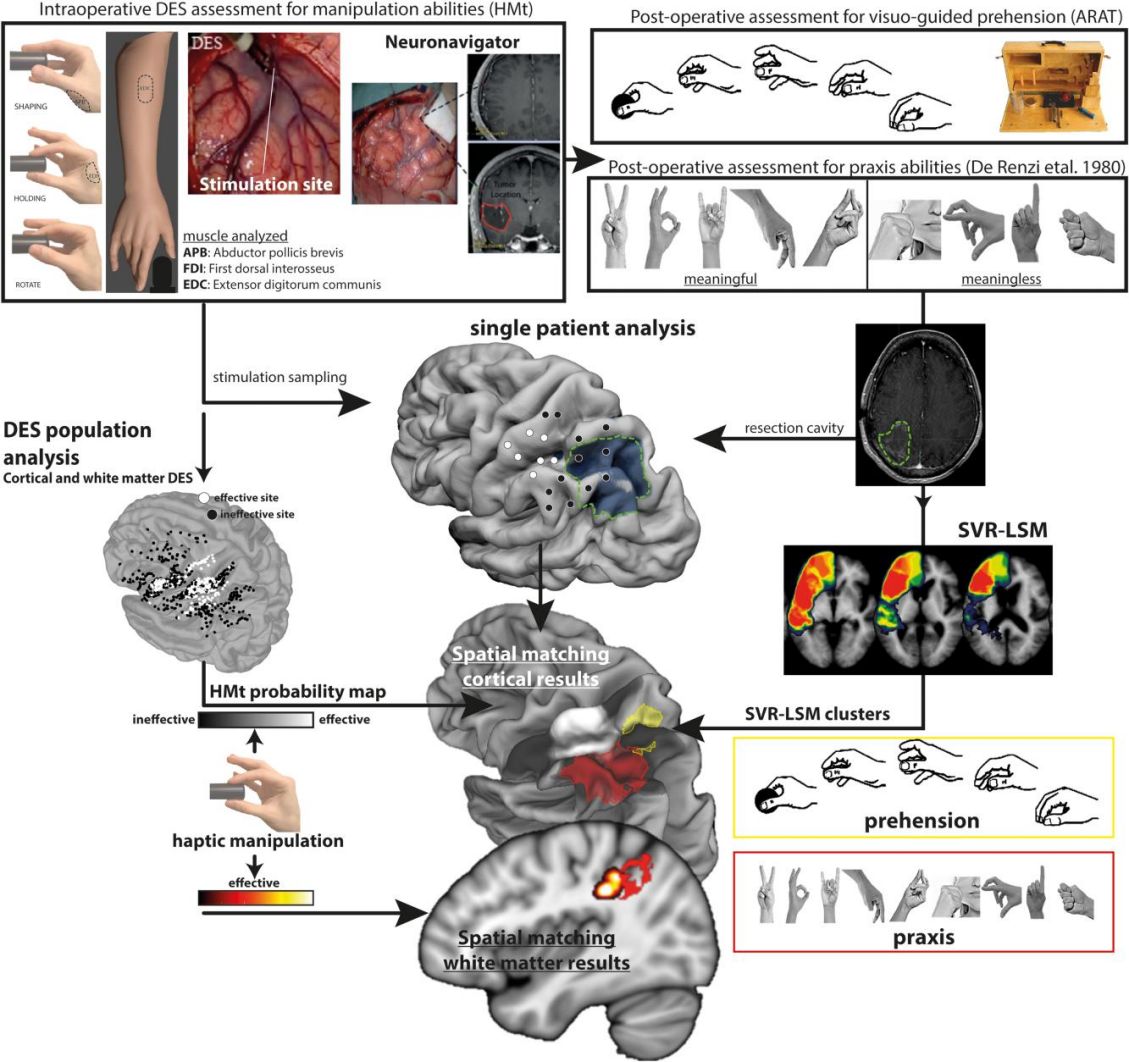
Schematic representation of the workflow of the study. ARAT = Action Research Arm Test; DES = direct electrical stimulation; HMt = hand manipulation task; SVR-LSM = support vector regression lesion symptom mapping.

All patients underwent standard brain mapping for language,^27^ motor,^28-31^ executive functions,^32,33^ visual field^34^ and manipulation abilities.^20-24^ Specifically, the areas crucial for manipulation abilities were assessed intraoperatively with a dedicated task, the HMt (Fig. 1). During the HMt, the patient was asked to grasp and continuously rotate a specific manipulandum-shaped object, with thumb and index opposition (precision grip). The HMt was performed in the absence of visual guidance. During HMt execution, the surgeon stimulated the cortical and subcortical areas required by the clinical needs with DES. During task execution, the hand behaviour and the electrical activity (EMG) of the intrinsic and extrinsic hand muscles were recorded and synchronized with DES. Intraoperative distinction between brain sites, where stimulation interfered (effective sites) or did not interfere (ineffective sites) with task execution, was based on both visual inspection of hand behaviour and the online monitoring of the EMG activity. An offline analysis of the EMG activity synchronized with video recordings of hand movement was then performed, allowing a more refined quantitative analysis (for further details see Fornia *et al*.^21,22^ and Viganò *et al*.^23,24^).

As well as the intraoperative assessment, a peri-operative evaluation of patients was performed before, at 7 days (early phase) and at 1-month after surgery using an extensive neuropsychological evaluation^35^ and specific tests to assess: (i) elementary sensory and motor disorders (objective neurological evaluation); (ii) visuo-guided prehension (reaching-to-grasping); and (iii) praxis abilities (imitation of intransitive gestures).

The preoperative assessment was needed to set the baseline condition of each patient (see inclusion criteria). The postoperative scores for prehension and praxis abilities of the selected patients were used in the support vector regression lesion symptom mapping (SVR-LSM) analysis to localize, among the brain areas surgically resected, those associated with lower scores in prehension and praxis performances. These results were spatially matched with the probability maps reporting the main cortical and subcortical intraoperative sites where DES interfered with manipulation abilities. See Fig. 1 for a summary of the methodological approach.

### Clinical assessments

#### Prehension assessment

The visually guided reaching-to-grasping abilities were assessed using the ARAT scale, which is composed of 19 items divided in four subscales: grasp, grip, pinch and gross movements.^36^ For the specific purpose of the study, the items were performed with the right upper limb (contralateral to the affected hemisphere). The ARAT was performed following standardized protocol and scoring.^37^ Instructions were read aloud and a visual demonstration for each item was provided. A time limit of 60 s was set to complete each task. The performance of each task was scored from 0 to 3: score = 3 was given when the task was performed correctly in <5 s (behavioural criteria outlined by Yozbatiran *et al*.^37^); score = 2 when the task was completed with overt abnormal hand and/or arm movements or with delay (from 6 to 60 s); score = 1 when the task was partially performed within the 60 s; score = 0 when none of the hand or arm movements required by the task were performed in 60 s. Relevant parameters adopted for the evaluation of the hand-arm performance of all sub-items were: (i) smoothness and precision of the reaching movement toward the object-space; and (ii) stability and congruency of the grip for each specific target. Since the focus of the ARAT, in the present study, was the clinical assessment of the visually guided reaching-to-grasping movements, the score obtained with gross movements was not included in the analysis, thus the maximum ARAT score (ARAT global score) was 48.

#### Ideomotor apraxia assessment

Praxis abilities were assessed with the De Renzi test^38,39^: patients were asked to imitate 24 intransitive gestures with the fingers, hand and arm. The task was performed with the contralesional right hand. During the test, the patients were asked to maintain the same body posture adopted during the ARAT while the examiners, sitting in front, showed each item to be imitated. To ensure full understanding of the instructions by the patients, the assessment started with a simple, test-unrelated, gesture to be imitated (rise the hand). Each gesture was presented up to three times and the performance scored from 3 to 0 depending on whether the execution was correct the first, second, third attempt or never. Among the 24 items, 12 were symbolic (meaningful) and 12 non-symbolic (meaningless) gestures. The total score was 72 (De Renzi global score). Notably, the imitation of the intransitive gesture relies on visual, proprioceptive and tactile feedback provided by contact between different fingers, between the hand/fingers and another body part or another external surface (see some examples in Fig. 1). In this regard, the tactile feedback is crucial for the correct execution of the gesture and its monitoring as well as for the correct object-grip during the visuo-guided prehension (ARAT).

### Image acquisition and lesion analysis

As part of the clinical routine, pre- and postoperative MRI was performed on a Philips Intera 3 T scanner and acquired for lesion morphological characterization and volumetric assessment.^28^ A post-contrast gadolinium T_1_-MPRAGE sequence was performed using the following parameters echo time: 2.75 ms, repetition time: 1600 ms, flip angle 9°, inversion time 900 ms; 176 slices; isotropic voxel size of 1 mm.

### Resection cavity tracing and spatial normalization

For each patient, the resection cavity was manually drawn on the postoperative volumetric T_1_-weighted images acquired at 5/7 days after surgery by L.F. with MRIcron software.^40^ This approach, with respect to the follow-up MRI (>1 month), had the benefit of avoiding interference with adjuvant treatments and is closest in time to the early postoperative praxis/prehension evaluation. Postoperative T_1_ and cavities of the patients were normalized to 1 × 1 × 1 mm resolution to Montreal Neurological Institute (MNI) space using the Clinical Toolbox implemented in SPM 12. In all patients, the normalization procedure was applied by using the enantiomorphic algorithm and lesion masking procedure. Since the study was based on performance assessments in the early postoperative phase, the cavity estimation was smoothed [full width at half-maximum (FWHM) 3 mm, threshold 0.05] for including a small amount of surrounding tissue. The rationale was that the tissue surrounding the resection cavity, spared from the resection, undergoes inflammation during the early postoperative phase that transiently impairs its function, possibly affecting behavioural performance. In addition, for each patient we also estimated the occurrence of ischaemic lesions in the postoperative diffusion weighted imaging (DWI) MRI sequence, in order to exclude patients with postoperative vascular diseases. Following the normalization procedure, the results were checked for each patient with CheckReg function in SPM 12.

### Lesion–symptom mapping

Multivariate LSM was performed using SVR-LSM^41^ implemented by DeMarco and Turkeltaub in a MATLAB-based toolbox.^42^ The analysis was performed by applying functionalities of the Statistic and Machine Learning Toolbox within MATLAB 2019b. Optimization of hyperparameters was performed via resubstitution loss and Bayesian optimization with 200 iterations and 5-fold cross-validation, as implemented in MATLAB (bayesopt) and recently applied by the authors of the MATLAB-base software and others group.^43-45^ In addition, the range for the optimized parameters was set following the range of C and Gamma suggested by Zhang *et al*.^41^ and more recently adopted by Wiesen *et al*.^46^ C range = 1–80, Gamma equivalent Sigma range = 0.1–30 (conversion was performed by using *function = gamma2sigma* available in SVR-LSM gui). A default Epsilon range was set. For each analysis and combination of parameters selected after the optimization procedure, both prediction accuracy and reproducibility were evaluated. Based on other studies using a similar procedure,^43,44^ we considered the LSM results reliable when showing accuracy ≥0.25 and reproducibility ≥ 0.85.

SVR-LSM was used to identify, in the early postoperative phase, significant voxels included in the resection cavities and/or around the borders associated with lower scores in the visuo-guided prehension (ARAT) and imitation of intransitive gestures (De Renzi) performance. Large lesions often result in more severe behavioural impairments, regardless of location, decreasing the specificity of the results. Thus, in the present study this aspect was controlled by applying the direct total lesion volume control (dTLVC), as implemented by Zhang *et al*.^41^ The resulting SVR-β values were thresholded at *P* < 0.005 and corrected with cluster size at *P* < 0.05, both based on 5000 permutations. In addition, continuous permutation-based family wise error (CFWE) correction was configured to permit 1.0 mm³ of false positives (desired *v* = 1 whole voxels) and accepted a family-wise error rate (FWER) of 0.05.

### Matching the SVR-LSM results and intraoperative direct electrical stimulation

#### Anatomical reconstruction of intraoperative direct electrical stimulation results

To match the postoperative SVR-LSM clusters associated with prehension and praxis lower scores with intraoperative cortical and white matter manipulation sites, the anatomical localization of each intraoperative effective site in each patient was needed.

##### Cortical sites

The exact position of the sites was reported on the 3D MRI (preoperative) cortical surface of each patient reconstructed with FreeSurfer by means of Brainstorm^45^ under the guidance of the flap-video and intraoperative coordinates from neuronavigation (BrainLab). Subsequently the MRI and site were co-registered to MNI space using Brainstorm and clinical toolbox in SPM 12.

##### Subcortical white matter sites

Included in this analysis were the effective stimulated sites located in the white matter below the sulci and/or grey matter, as reported by intraoperative coordinates in native space and surgical flap. During postoperative reconstruction, the site was drawn on the preoperative axial volumetric T_1_ as spherical region of interest (ROI) (6 mm diameter, similar to the resolution of the bipolar probe) based on image and related native coordinates acquired with neuronavigation system. The localization of the site was also verified using the postoperative T_1_ as reference. As the effective sites were used as functional borders to stop the resection, the edge of the resection cavity represents an optimal landmark to confirm the site positioning. To this aim, in each patient, the stimulation sites, the preoperative T_1_ and the postoperative T_1_ were co-registered to the MNI space by means of the Clinical Toolbox implemented in SPM12.

The accuracy of each co-registration was visually confirmed using the SPM12 CheckReg function. Finally, the anatomical localization of each site in each patient was confirmed by the first operating surgeon (L.B.).

To investigate whether the effective sites clustered in specific subsectors, a modified in-house version of probability kernel density estimation (PDE analysis) implemented in MATLAB was applied (see Fornia *et al*.^21,22^ for details regarding PDE for cortical sites and Viganò *et al*.^24^ for PDE for subcortical sites).

## Results

In all the enrolled patients the impact of basic (primary) motor disorders in performance of the ARAT and De Renzi tests was excluded by enrolling patients with MRC score >4 and with score = 3 in grasping the ‘heaviest’ wooden cube (10 cm) of the ARAT. The level of whole handgrip strength and somatosensory feedback needed to execute the task excludes the occurrence of primary somatosensory and motor deficits possibly affecting ARAT and De Renzi tasks. Based on these criteria, seven patients initially recruited were not included in the final analysis.

The results presented here will report: (i) ARAT and De Renzi clinical scores distribution; (ii) SVR-LSM results; (iii) intraoperative DES results; and (iv) spatial matching between SVR-LSM and DES.

### ARAT and De Renzi clinical scores

#### ARAT

**Figure 2:**
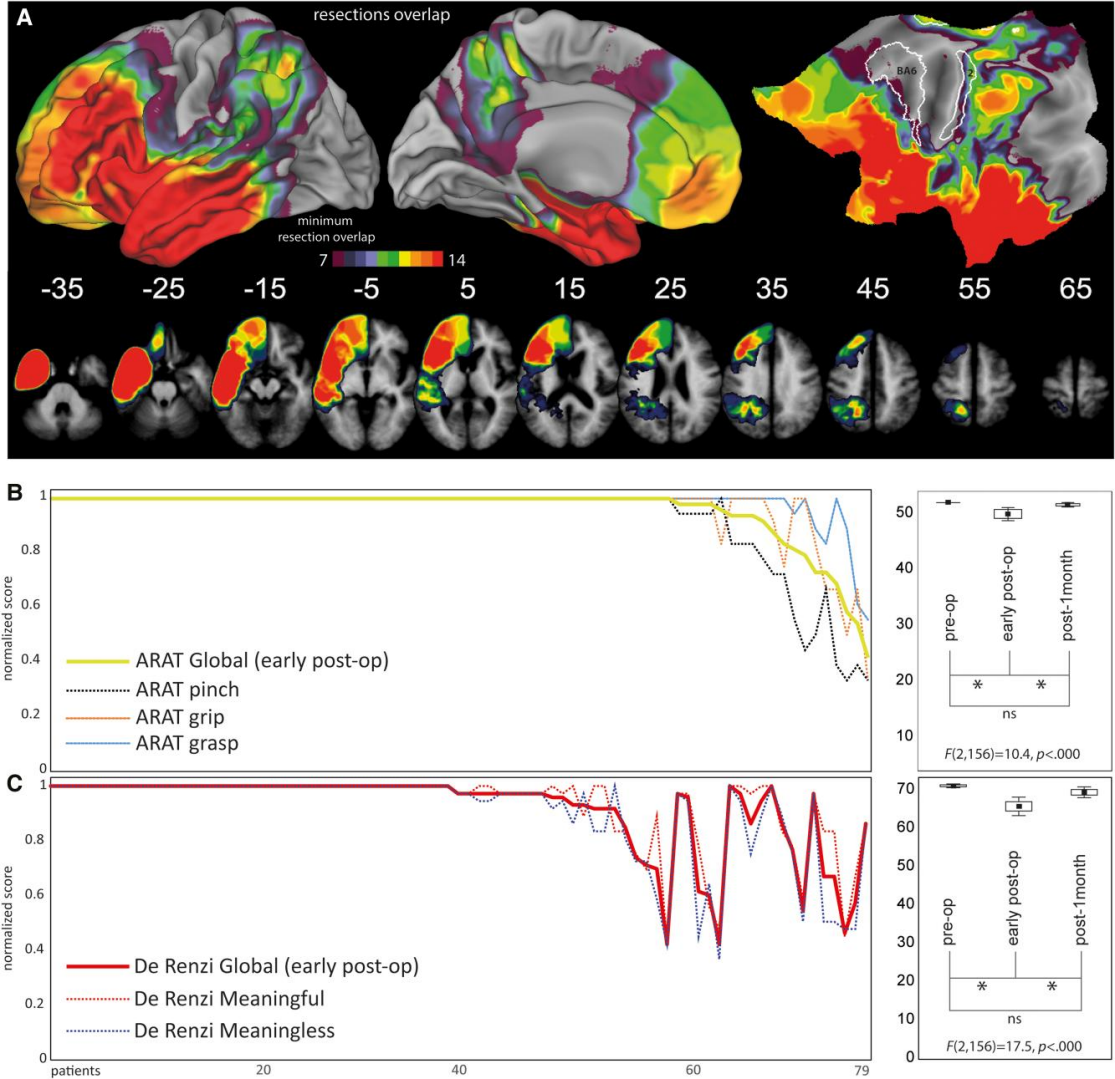
**Resection cavities overlap and scores.** (**A**) Overlapping map of patients’ resection cavities; (**B**) distribution of De Renzi and (**C**) ARAT scores and pre/post-surgery statistical results. ARAT = Action Research Arm Test; ns = not significant.

Since ARAT scores are a continuous measure, without categorical cut-off scores, patients were ranked based on the number of items showing a performance decrease with respect to the preoperative assessment. Preoperative phase: none of the patients showed observable deficits. Early postoperative: 19 out of 79 patients reported a lower score in the early postoperative phase subdivided as follows: (i) 10 out of 79 patients with a score decrease only in the pinch subscale (12.6% of patients, average global score 45.2, range 38–46); (ii) five out of 79 patients with a score decrease in pinch-grip items (6.3% of patients, average global score 38.2, range 33–42); and (iii) four patients with a score decrease in grasp-grip-pinch (5% of patients, average global score 27.25, range 20–35). Postoperative 1 month: at population level, the ARAT global score significantly improved compared to the early postoperative phase with no significant difference with the preoperative phase (Fig. 2B: ARAT scores distribution and statistics).

#### De Renzi

Preoperative phase: none of the included patients had a pathological score and only three patients showed a borderline global score. Early postoperative: 11 out of 79 patients global score fell below the cut-off (13.9% of the patients, cut-off <53, average 41.5, range 30–51) while six patients were borderline (7.6%, cut-off 53–62, average 59.2, range 53–62). The remaining 62 patients scored above the cut-off (78.5%, average 70.9, range 63–72). Postoperative 1 month: at population level, De Renzi global score significantly improved compared to the early postoperative phase with no significant difference with the preoperative phase. These results, coherent with a previous study from our group,^20^ confirm the transitory nature of the early postoperative outcome (Fig. 2C: De Renzi scores distribution and statistics). See Supplementary Table 1 for clinical details.

#### De Renzi and ARAT scores correlation

To assess whether the De Renzi and ARAT early pos-operative scores are correlated, a Spearman correlation was performed on the global scores. At population level, ARAT and De Renzi global scores significantly correlated (Spearman correlation r = 0.557, *P* < 0.05). However, among ARAT items, the regression analysis showed that only score of the pinch item significantly predicted a decline in the De Renzi performance [predictors: grasp, grip, pinch; dependent variable: De Renzi global score; *F*(3,75) = 9.74, *P* < 0.000, R^2 adjusted^ = 0.25; grasp *F* = 1.72, *P* = 0.19, beta = −0.22; grip *F* = 2.3, *P* = 0.13, beta = 0.306; pinch *F* = 6.17, *P* = 0.015, beta = 0.408].

### SVR-LSM results

#### Localization of surgical cavities

The SVR-LSM analysis was restricted to the minimum overlap of seven patients (∼9% of the whole sample). The minimum overlap involved the frontal areas [excluding Brodmann area (BA)4–BA6], parietal lobe (excluding area 3a, 3b, 1 and marginally including area 2), the temporal lobe, the insular cortex and adjacent opercular regions (Fig. 2A). Due to the significant correlation between prehension and praxis global scores, we performed SVR-LSM analysis with and without covariate to investigate specific (covariates results) and common (overlap of non-covariate results) voxels for prehension and praxis. SVR-LSM analysis was performed for both early postoperative and 1-month De Renzi and ARAT scores. We report only the early postoperative results, since the prediction accuracy and reproducibility values for the SVR-LSM at 1 month fell below the threshold considered (accuracy <0.25, reproducibility <0.85).

#### Prehension abilities: De Renzi covariate

**Figure 3:**
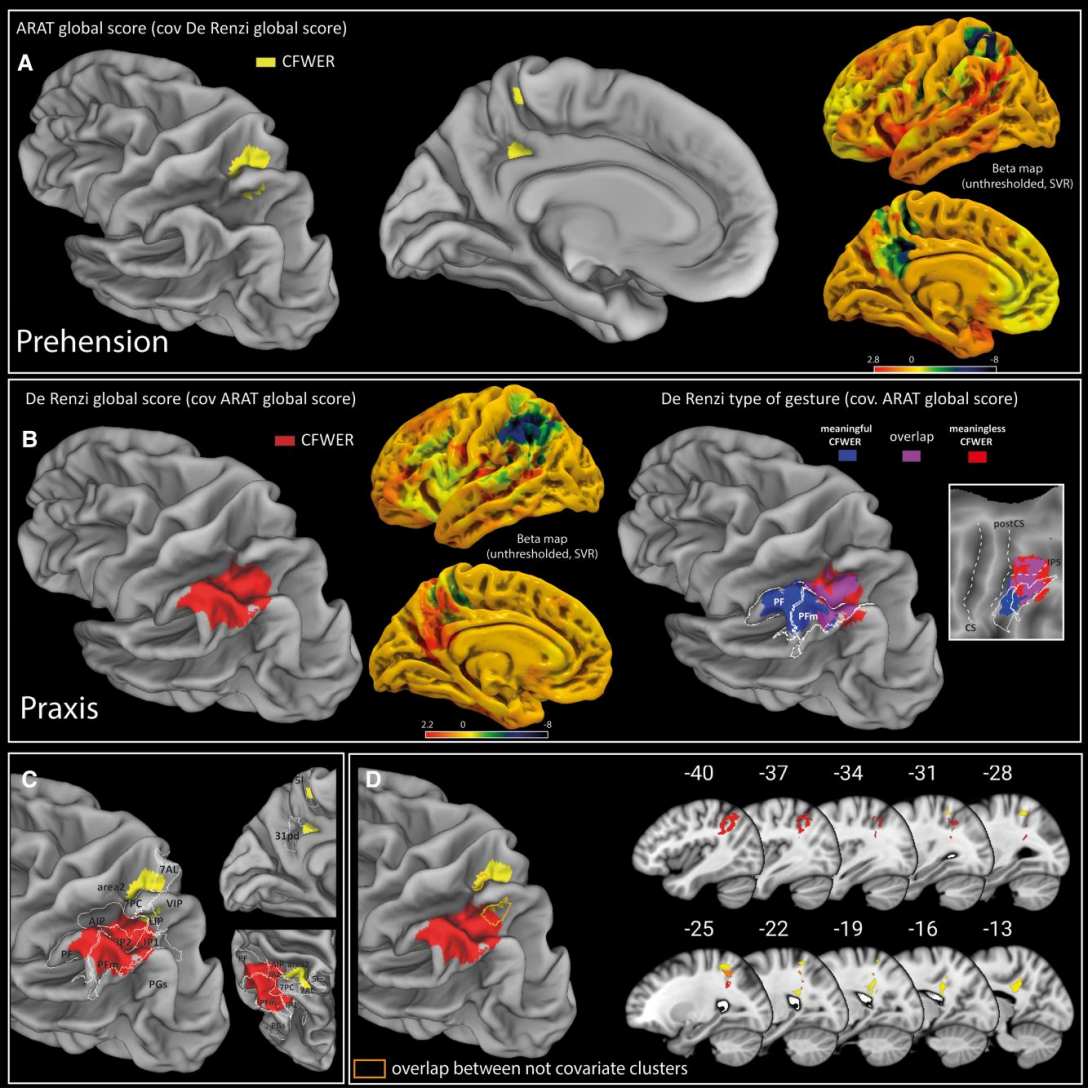
**SVR-LSM results.** (**A**) SVR-LSM results for the ARAT global score covariate with the De Renzi global score. (**B**) SVR-LSM results for the De Renzi global score covariate with the ARAT global score and SVR-LSM results for meaningless and meaningful gestures both covariate for the ARAT global score. (**C**) Overlap between ARAT and De Renzi covariate CFWER clusters with HCP-MMP1 parietal regions. (**D**) Overlap between ARAT and De Renzi CFWER covariate results and common region resulting from overlap of non-covariate CFWER results. ARAT = Action Research Arm Test; CFWER = cluster-level family-wise error correction; SVR-LSM = support vector regression lesion symptom mapping.

Results showed that a decrease in the visuo-guided object-prehension abilities was associated with CFWER cluster involving: (i) the superior parietal lobe (7AL, 7PC, 5L and LIP); and (ii) the precuneus (31pd) (Fig. 3A–C). Hyperparameters: cost/box constraint = 77, sigma/kernel scale = 1.54, epsilon = 1.9; prediction accuracy = 0.41, reproducibility index *r* = 0.86.

#### Praxis abilities: ARAT global score covariate

Results showed that a decrease in the imitation of intransitive gesture was associated with a CFWER cluster involving: (i) the intraparietal (anterior intraparietal [AIP], IP2); and (ii) the inferior parietal lobe (PF/PFm) (Fig. 3B and C). Hyperparameters: cost/box constraint = 75, sigma/kernel scale = 1.25, epsilon = 0.1; prediction accuracy = 0.54, reproducibility index *r* = 0.86.

#### Meaningful versus meaningless praxis gestures: ARAT global score covariate

Results showed that although the two CFWER clusters overlapped within the intraparietal sulcus (IPS), meaningful gesture clustered in a wider area in the inferior parietal lobe (IPL) with respect to meaningless gesture, including mainly PF (Fig. 3B). Hyperparameters meaningful: cost/box constraint = 79, sigma/kernel scale = 1.29, epsilon = 1.8; prediction accuracy = 0.53, reproducibility index *r* = 0.87. Hyperparameters meaningless: cost/box constraint = 79, sigma/kernel scale = 1.13, epsilon = 2.49; prediction accuracy = 0.48, reproducibility index *r* = 0.85.

#### Common region: overlap between ARAT and De Renzi not covariate results

Results showed that common voxels were found mainly within the dorsal bank of the intraparietal sulcus and adjacent dorsal postcentral sulcus (Fig. 3D). Hyperparameters ARAT: cost/box constraint = 30.6, sigma/kernel scale = 1, epsilon = 0.1; prediction accuracy = 0.45, reproducibility index *r* = 0.85. Hyperparameters De Renzi: cost/box constraint = 45.3, sigma/kernel scale = 1.23, epsilon = 0.38; prediction accuracy = 0.59, reproducibility index *r* = 0.86.

### Intraoperative direct electrical stimulation results

Previous studies from our group have reported evidence that intraoperative DES delivered on specific premotor^21^ and parietal areas^22^ as well as on frontal white matter^24^ affects the performance of tasks requiring hand-object manipulation (HMt). Since the SVR-LSM analysis performed in the present study highlighted significant clusters only in the parietal lobe, the spatial matching analysis between lesion results and intraoperative DES results was constrained to the intraoperative data recorded within the parietal lobe^22^ at cortical and, a novel finding here, at subcortical level within the parietal white matter. Cortical and subcortical data together allow a more comprehensive spatial matching with SVR-LSM results.

#### Cortical results

**Figure 4:**
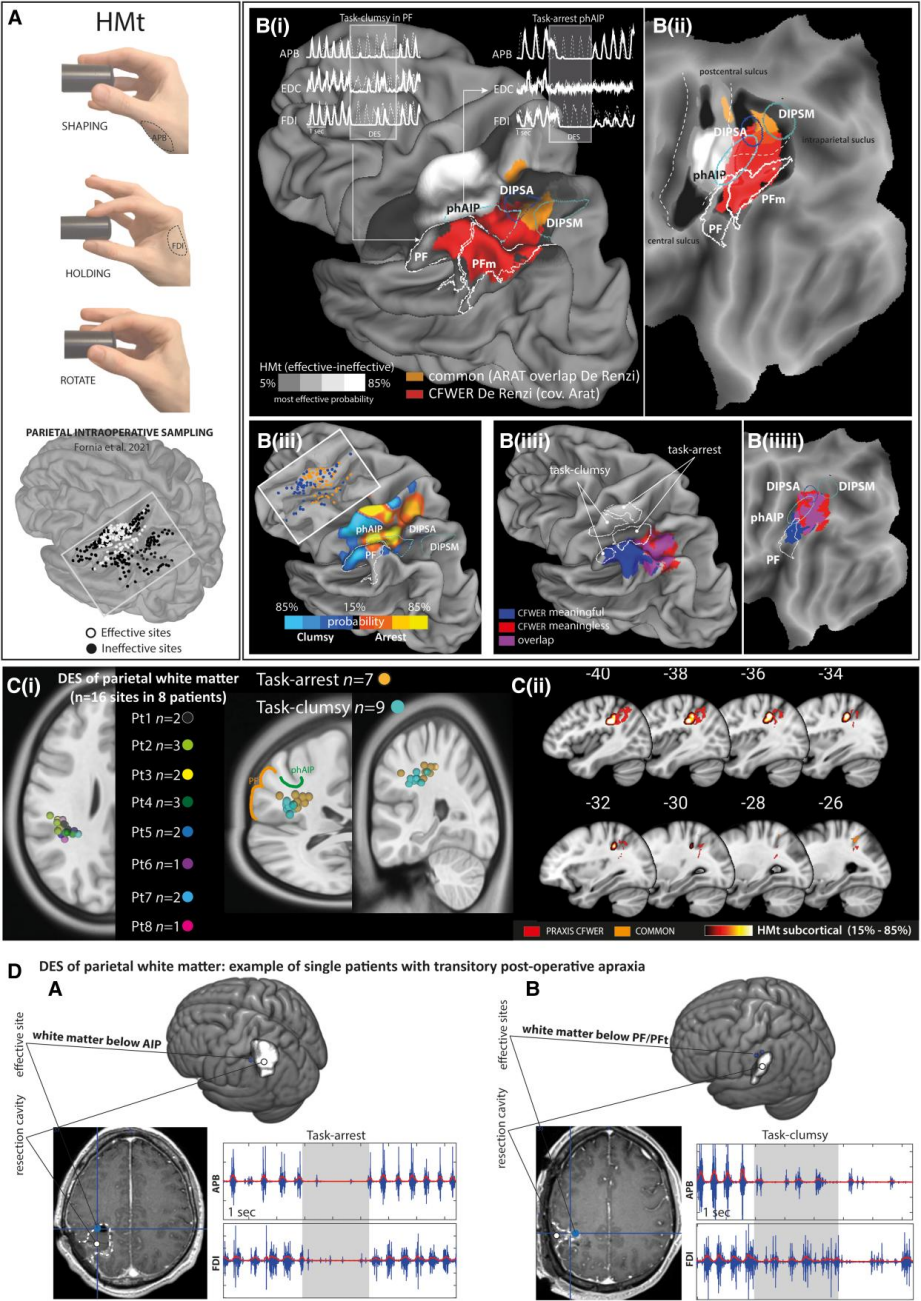
**Spatial matching between DES and SVR-LSM results.** (**A**) Hand manipulation task (HMt) and sampling of parietal stimulation from Fornia *et al*.^21^ [**B**(**i, ii**)] Co-localization between HMt probability density estimation (effective areas in white and ineffective areas in black), praxis cluster (red) and prehension-praxis common region (orange). The *upper* part of [**B**(**i**)] shows examples of EMG interference patterns evoked by parietal DES of phAIP and PF. [**B**(**iii**)] HMt probability maps showing the parietal region associated with different EMG-interference patterns (task-clumsy versus task-arrest) regardless of ineffective sites. [**B**(**iiii**)] Co-localization between HMt task-arrest and clumsy pattern probability with meaningful (blue), meaningless (red) gestures CFWER (covariate ARAT) and posterior parietal regions (phAIP, DIPSA, DIPSM, PF). [**C**(**i**)] Anatomical localization of effective sites recorded within the parietal white matter. [**C**(**ii**)] Probability density estimation of HMt effective sites within the white matter and their co-localization with praxis CFWER (covariate ARAT). (**D**) Example of two patients showing transient postoperative apraxia: (**A**) the effective site was located in the white matter below the AIP and evoked a task-arrest pattern; (**B**) the effective sites were located in the white matter below the PF and evoked task-clumsy patterns. ARAT = Action Research Arm Test; CFWER = cluster-level family-wise error correction; DES = direct electrical stimulation; DIPSA = dorso-anterior intraparietal sulcus; DIPSM = dorso-medial intraparietal sulcus; phAIP = putative homologue of anterior intraparietal, AIP; SVR-LSM = support vector regression lesion symptom mapping.

These results were previously published.^21^ In brief, intraoperative DES of specific parietal sectors interfered with performance of HMt by disrupting recruitment of the the hand muscles. Probability density estimation, obtained by contrasting effective sites (*n* = 111) with ineffective sites, highlighted significant responsive clusters in the post-central gyrus (somatosensory fingers representation), the putative human homologue of monkey AIP (phAIP^47^) and, more marginally, the anterior PF/PFt within the IPL [Fig. 4A and B(i-ii)]. Within the posterior parietal cortex, DES effect on HMt ranged from an abrupt arrest (task-arrest) mainly reported within phAIP, to a lack of finger coordination (task-clumsy), mainly reported within anterior IPL (PF), both associated with different degrees of muscle suppression [Fig. 4B(iii)].

#### White matter results

Considering the eight patients showing effective sites in the deep white matter of the posterior parietal cortex, 16 effective sites were localized (according to the patient native space) in the white matter below the fundus of rostral IPS and postcentral sulcus, broadly corresponding to the white matter below phAIP and PF/PFt. Task-arrest (*n* = 7) responses were mainly found below phAIP while task-clumsy (*n* = 9) were adjacent to the white matter below PF [Fig. 4C(i)], coherently with cortical distribution.

#### Matching praxis and manipulation cortical and subcortical sites

The spatial matching analysis showed that intraoperative manipulation-sites and praxis-related clusters co-localized within rostral IPS and IPL regions. More specifically:

1. Within rostral IPS, the intraoperative manipulation-sites clustered within the anterior part of phAIP, while praxis-related voxels at the transition between phAIP and dorso-anterior intraparietal sulcus (DIPSA) [Fig. 4B(i–ii)].
2. Within the rostral IPL, despite the lower level of probability, intraoperative manipulation sites clustered in anterior PF, while praxis-related voxels at the transition between the PF and PFm [Fig. 4B(i,ii)].
3. The matching obtained at cortical level was specular at subcortical level [Fig. 4C(i,ii)].
4. The anterior IPS was associated with both meaningless and meaningful gestures, while anterior IPL was associated with meaningful gestures. Parallel to this distinction, the manipulation sites within the rostral IPS (phAIP) and IPL (PF) showed different features of motor impairment induced by DES during the HMt, task-arrest and clumsy, respectively [Fig. 4B(iii,iv) and 4C(i)].

## Discussion

In the present study, we used complementary causal techniques in brain tumour patients. Postoperative lesion mapping was used to investigate prehension and praxis-related regions, and intraoperative DES to investigate object manipulation-related regions. A spatial matching between the results of the two techniques was employed to investigate the anatomo-functional relationship between the neural substrates subserving praxis abilities (imitation of intransitive gestures) and the phylogenetically old building blocks subserving object-oriented actions (prehension and object-manipulation).

Studies in stroke patients significantly contributed to outline the current theoretical, anatomical and clinical framework in the field of praxis-related disorders. However, several aspects related to the different aetiology and clinical outcome prevent a strict comparison between LSM results collected in stroke and brain tumour patients.^48^ In this regard, brain tumour is a focal lesion and the resulting resection cavity following the brain mapping technique is well identifiable and functionally delimited.

In a different frame, fMRI studies also provided important insight in the field of praxis movements. However, fMRI data were correlational by nature and did not allow us to investigate the causal functional role of the different nodes belonging to the PRN. In this regard, the LSM and DES are historically considered the gold standard for causal mapping of human brain functions allowing us to draw causal inferences about the role of a specific region with respect to the investigated function, a crucial aspect for translating knowledge into therapeutic targets.^49^

However, despite the aforementioned advantages, both LSM and DES have limitations in the present clinical context, in particular the preoperative brain functional reorganization and the morphological displacement due to tumour mass. Our group adopted specific patient inclusion criteria for reducing the impact of tumour displacement^22^ and for estimating the quality of the coregistration.^24^ Despite these criteria not being able fully prevent the impact of such variables on results, the spatial matching between the intraoperative DES and postoperative LSM results was in line with the clinical aim of the intraoperative brain mapping, supporting a good reliability of the results obtained by integrating different methods, at least within the sample of patients enrolled in this study. Finally, based on recent guidelines,^50^ another potential limitation of the present study was the small sample of patients required to optimally model voxel-wise lesion location in SVR-LSM. However, considering that brain tumour is a rare pathology and the patient inclusion criteria adopted for the aim of the present study, the resection cavities equally covered frontal, parietal and temporal areas, allowing us to causally test the main nodes of the dorso-dorsal and dorso-ventral pathways.

### The dorso-ventral stream is specifically implicated in praxis abilities

The first result emerging from this study was the higher relevance of the left posterior parietal lobe, with respect to prefrontal or temporal, in the onset of ideomotor apraxia, in agreement with initial observations.^51^ Within the parietal lobe, neuroimaging and lesion studies provided evidence of the involvement of several dorso-dorsal and dorso-ventral parietal sectors in the imitation of intransitive gestures.^52^ The present study, investigating ideomotor apraxia by using prehension performance as covariate, clearly showed that specific ideomotor praxis deficits were associated with parietal sectors included in the dorso-ventral stream rather than in the dorso-dorsal stream. Specifically, the borders of resections adjacent to rostral IPS and IPL are associated with a transient impairment in imitation of intransitive gestures, while more dorsal resections involving the anterior superior parietal lobe and the precuneus cause a specific impairment in visually guided object-prehension. Notably, the absence of a quantitative kinematic-based approach to prehension movements prevent, in the present study, the investigation of micro-features of movement. However, coherently with our finding, these areas are classically associated with optic ataxia, a high order deficit in reaching visual goals, hand preshaping and on-line correction during reaching.^53-55^

The dissociation found in the present study is in agreement with evidence suggesting that, although highly coordinated, dorso-dorsal and dorso-ventral pathways play distinct roles in hand actions. In this regard, converging evidence has shown that the dorso-dorsal system, also called ‘Grasp system’, processes visual-related object physical features for the purpose of prehensile action, while the dorso-ventral stream, also called ‘Use system’, is involved in the long-term storage of the particular skilled actions associated with familiar objects.^56^ Coherently, the two systems are connected differently with temporal (ventral stream) areas and are involved in the extraction of different type of object affordances. Accordingly, the invariant object features, i.e. stable affordances, emerge from the slow ‘offline’ processing of the visual information based on memorized object knowledge taken over by the dorso-ventral pathway. Conversely, changing or temporary object physical features, i.e. variable affordances, emerge from the fast online processing of visual information during actual object interaction, mainly in charge to the dorso-dorsal pathway.^57^ The present results may extend these distinctions showing that specific parietal nodes within the dorso-ventral pathway are also crucial for imitation of intransitive gestures. In this regard, the gesture execution occurs through its observation and recently it has been proposed that visual encoding of other’s actions, i.e. social affordances—conceived as the variety of action possibilities offered to an individual by another’s behavior—exist alongside object affordances. This hypothesis extends the concept of affordances from inanimate object to the other’s action.^58^ In line with this view, the present results may suggest that the dorso-ventral pathway not only encodes stable (complex) affordances related to purposeful interaction with objects, but may also extract social affordances via the observation of the gesture to be imitated. The latter mechanism may be crucial in ideomotor apraxia, possibly favouring the visuomotor conversion of the observed gesture.

Furthermore, although the two streams process distinct action features, the IPS emerged as a convergence zone.^57^ This finding is coherent with the results of the present study, which points to the dorsal bank of the IPS, mainly corresponding to the transition between dorso-anterior and medial intraparietal sulcus (DIPSA and DIPSM), as a potential common region. This fits with the positive correlation found between prehension and praxis scores, which suggested that, to some extent, the two pathways work along a functional continuum rather than in dichotomous way. In this regard, the existence of this intraparietal hub may subserve common functional aspects and/or the exchange of information between dorso-dorsal and dorso-ventral streams,^57^ possibly contributing at the extremely flexible use of our hand sensorimotor repertoire, from concrete action specification to abstract action goals.^59^ Interestingly, the regression analysis showed that the sole ARAT item significantly predicting the De Renzi performance was the pinch. The pinch item requires a higher level of dexterity with respect to the other ARAT items and often requires an ‘unusual’ or ‘less functional’ grip posture (i.e. to execute a precision grasping with thumb-ring/thumb-middle finger opposition). Although the interpretation of this result is challenging, it might suggest that, when the required hand action is less consolidated in our daily sensorimotor repertoire, an efficient communication between streams via intraparietal hubs could be crucial for its implementation.

### Parietal lobe hosts distinct praxis route and sensorimotor processes: a comparative perspective

As previously reported,^22^ DES delivered during the HMt onto phAIP evoked an abrupt arrest (task-arrest) while on PF, it evoked a loss of coordination of finger (i.e. task-clumsy). We suggested that the task arrest might reflect the ‘transient impairment’ of a parietal sector shaping, with a relatively direct access, the motor output. Conversely, the clumsy pattern may reflect the ‘transient impairment’ of a parietal sector hierarchically far with respect to the cortical motor output. Overall, we suggest that the different impairments may ultimately arise from a different role of rostral IPS and IPL regions in shaping hand-motor output.

Paralleling the anatomical distribution of the different effects of DES on HMt, the LSM results showed that the parietal sectors adjacent to phAIP and PF were associated with different deficits in the imitation/execution of gestures. Resections involving the rostral IPS affected the imitation of both meaningless and meaningful gestures (no gesture type selectivity), while resections involving the rostral PF complex mainly affected the imitation of meaningful gestures (gesture type selectivity), in line with the evidence of a dissociation between the two types of intransitive gestures.^60,61^ Taken together, the co-localization between intraoperative DES and LSM results suggests that the different motor impairments evoked by DES within phAIP and PF may reflect different sensorimotor processes, possibly subserving different pathways for gesture imitation. In this regard, gesture imitation is indeed subserved by two pathways: the direct pathway involved in the execution of the observed gesture regardless to its content; and the indirect pathway controlling reproduction of gestures through access to their meaning in the semantic memory.^62^ In this light, our results suggest that the former may take place within the rostral IPS (cortical area hosting task-arrest and no gesture type selectivity), while the latter may take place within the PF complex (cortical area hosting task-clumsy and gesture type selectivity).

#### The rostral intraparietal sulcus and the direct pathway

The co-localization within rostral IPS of DES-related effects on manipulation (the arrest effect) and voxel associated with imitation of the observed gestures (no gesture type specificity) might suggest that the integrity of this region could be crucial for both the motor implementation and the visuomotor conversion of the observed gesture.

In this regard, it has recently been shown that monkeys and human rostral IPS hosts neurons selective for observed manipulative actions claimed to support a stable readout of the observed actions across visual formats.^58,63-65^ Despite the impressive similarities between human and monkey results, neurons in humans showed a greater invariance and generalization across viewpoint compared to those in monkeys, including responses to reading action verbs. The greater invariance and generalization in humans may reasonably point to the human IPS as the encoder of a wide variety of actions formats, including non-manipulative actions, such as the processing of the observed intransitive gesture. This hypothesis is coherent with fMRI studies in healthy subjects, showing that both transitive and intransitive gestures are processed within the left-lateralized praxis representation network, including the rostral IPS.^10,66,67^ The selectivity for the observed manipulative actions within the rostral IPS, essential for action planning during social interaction and inter-individual coordination, is suggested to work in parallel with the neural population involved in the sensorimotor transformation for object-oriented action.^58^ Based on this premise, we may hypothesize that praxis-significant voxels within phAIP/DIPSA transition could reflect the role of this sector in the visual processing of the observed gesture. Moreover, since the DES of rostral phAIP affected the hand manipulation motor output evoking task-arrest responses, we might speculate that visual information of the observed gesture may be exploited by phAIP and its connectivity with premotor areas for the motor implementation of the gesture itself. This functional organization fits with the supposed role of phAIP and DIPSA as motor and visual sector of monkey AIP, respectively.^47^

Despite being obtained by the complementary use of different tasks (HMt and De Renzi) testing distinct action features (transitive versus intransitive), these results could support the idea of a direct pathway within the rostral IPS subserving visuo-motor conversion during gestures observation/imitation. Notably, this pathway seems to rely prevalently on phylogenetically old rostral IPS nodes, with its main hub within phAIP, a core area belonging to the lateral grasping network (LGN^3^), originally described in monkeys. The LGN belongs to the dorso-ventral pathway and is considered a cognitive interface for hand actions.^4^ Our finding that the co-localization of manipulation and praxis voxels within the human rostral IPS strengthens the hypothesis that the anatomo-functional features of the monkey’s LGN fostered the cognitive upgrade of the dorso-ventral pathway further subserving the unique human praxis repertoire. Moreover, the dual role in hand-object and hand-gesture oriented actions could reasonably explain why the preservation of the rostral IPS during intraoperative mapping with the HMt, a task not actually directly assessing praxis functions, resulted in prevention of permanent ideomotor apraxia symptoms.

### The inferior parietal lobe and the indirect pathway

Conversely, according to LSM results, lesions within the PF complex critically impair imitation of meaningful gestures, identifying this region, with access to semantic knowledge, as a key structure in the indirect pathway for gesture imitation. Interestingly, DES of the IPL/PF and its surrounding white matter alters motor execution by evoking task-clumsy responses, hypothesized to reflect a remote access to the motor output with respect to task-arrest. In light of this evidence, we may speculate that the properties of the parietal clumsy region, the PF complex, may subserve the integration and gating of conceptual and semantic knowledge into the pragmatic sensorimotor workflow. This hypothesis is in agreement with theories suggesting that meaning or conceptual knowledge would emerge from interactions between multimodal areas and the pathways processing motor information.^68^ The rostral PF complex may favour this connection. In this frame, the overall IPL is considered a semantic hub active during semantic processing of cross-modal spatial and temporal configurations,^69^ thus we hypothesize that PF-related pathways may act as a passageway integrating this information from parietal and temporal high-order multimodal areas, into the sensorimotor workflow taken over by rostral IPS.

## Conclusion

Overall, the anatomo-functional interaction between the rostral IPS and IPL areas likely represents the neural mechanism by which cognition shapes sensorimotor processing, ultimately promoting the unique human hand actions repertoire.

From a clinical perspective, our results suggest that the preservation of this mechanism is crucial for avoiding long-standing ideomotor apraxia in brain tumour patients. Preservation of these dorso-ventral parietal regions was possible thanks to the DES applied during the HMt, which allowed us to identify the functional borders of these areas at a cortical and subcortical level, as clearly showed by the spatial matching between the DES and LSM results. Therefore, the transient nature of the symptom may be due to the inflammatory state of the tissues preserved at the edge of the resection and/or to a partial impairment of its functioning. Regarding the latter point, we could not exclude that, since their role as hub regions within the PRN, these areas may host an extended connectivity, which may promote functional compensatory mechanisms.^70^

To summarize, the present results showed a functional dissociation between dorso-dorsal and dorso-ventral streams and within the dorso-ventral stream. First, it identified the existence of a parietal dorso-lateral functional continuum subserving the transition from transitive object-oriented actions (dorso-dorsal pathway) to intransitive praxis gestures (dorso-ventral pathway), with specific rostral IPS sectors possibly working as a convergent zone and regulating the flow of information between streams. Moreover, within the dorso-ventral stream, our results showed a further dissociation between the role played by rostral IPS (mainly phAIP/DIPSA) and rostral IPL (mainly PF) in the type of gesture to be imitated (meaningless versus meaningful), to some extent mirroring the anatomo-functional distinction between object-manipulation and object (tool)-use.^5,71^ Notably, the DES applied to these parietal regions evoked a different type of motor impairments during the HMt execution, furthermore suggesting that these sectors may subserve distinct pathways for gesture imitation (direct versus indirect) via different hand-related somatomotor processes.

Finally, these areas—in addition to be part of the PRN and the LGN in human and non-human primates, respectively, in particular IP2 and PFm areas—are also core regions within the multiple demand network (MDN^72^). Since its definition, the MDN is implicated in a range of cognitively demanding tasks and appears central to intelligent action.^73^ Taken together, this evidence highlights the multidimensional nature of the human praxis abilities and the importance of sensorimotor substrates adjacent and/or interleaved with multimodal areas in translating both gesture-related visual information and conceptual knowledge into a coherent motor representation.

Regarding limitations, a potential bias for the present results could be the lack of systematic control conditions allowing us to quantify the integrity of the various modalities of sensory feedbacks exploited by the tasks, in order to exclude them as confounding factor. This is a relevant issue, since intraoperative HMt and postoperative tasks rely on sensory-guided modalities not completely overlapping. In this regard, this bias was qualitatively overcome by excluding patients that showed clinically overt basic visual and tactile deficits during neurological assessment. However, this procedure may not be exhaustive since the posterior parietal lobe hosts high-order sensory modalities crucial for the sensorimotor guidance of the three tasks. To reduce this confounding aspect, we used the ARAT score as covariate in LSM analysis for praxis functions. Since the ARAT execution relys on both somatosensory (shared with the HMt and praxis tasks) and visual-guidance (shared only with the praxis tasks), its use as covariate for investigating praxis abilities allowed us to isolate task-specific voxels for the imitation of the observed gesture. Finally, these methodological aspects allowed a spatial matching between ARAT versus De Renzi and HMt versus De Renzi, mainly reflecting intrinsic task features.

## Supplementary Material

awad316_Supplementary_Data

## Data Availability

The data that support the findings of this study is available from the corresponding author, upon reasonable request.
