# Supplementary material for: The parietal architecture binding cognition to sensorimotor integration: a multimodal causal study
Source: Brain. 2023 Sep 16;147(1):297–310. doi: 10.1093/brain/awad316 (PMC10766244; doi:10.1093/brain/awad316)
Supplement: awad316_Supplementary_Data [file awad316_supplementary_data.pdf]

Supplementary Table 1 Scores and Clinical Data

Pt	ARAT early post-op				De Renzi early post-op			De Renzi 1month post-op	De Renzi pre-op	ARAT 1month post-op	ARAT pre-op		
	grasp	grip	pinch	global score	meanful	meanless	global score	global score	global score	global score	global score	GRADE	Localization
**Pt1**	18	12	18	48	36	36	72	72	72	48	48	other	P
**Pt2**	18	12	18	48	36	36	72	72	72	48	48	HGG	F
**Pt3**	18	12	18	48	36	36	72	72	72	48	48	LGG	P
**Pt4**	18	12	18	48	36	36	72	72	72	48	48	HGG	F
**Pt5**	18	12	18	48	36	36	72	72	72	48	48	LGG	F
**Pt6**	18	12	18	48	36	36	72	72	72	48	48	other	P
**Pt7**	18	12	18	48	36	36	72	72	72	48	48	HGG	T
**Pt8**	18	12	18	48	36	36	72	72	72	48	48	HGG	F-T
**Pt9**	18	12	18	48	36	36	72	72	72	48	48	HGG	T-P
**Pt10**	18	12	18	48	36	36	72	72	72	48	48	LGG	T-P
**Pt11**	18	12	18	48	36	36	72	72	72	48	48	HGG	T
**Pt12**	18	12	18	48	36	36	72	72	72	48	48	LGG	T-P
**Pt13**	18	12	18	48	36	36	72	72	72	48	48	LGG	T-P
**Pt14**	18	12	18	48	36	36	72	72	72	48	48	HGG	T
**Pt15**	18	12	18	48	36	36	72	72	72	48	48	LGG	T
**Pt16**	18	12	18	48	36	36	72	72	72	48	48	HGG	T-P
**Pt17**	18	12	18	48	36	36	72	72	72	48	48	HGG	T
**Pt18**	18	12	18	48	36	36	72	72	72	48	48	HGG	F

Supplementary Table 1 Scores and Clinical Data

**Pt19**	18	12	18	48	36	36	72	72	72	48	48	HGG	F-T
**Pt20**	18	12	18	48	36	36	72	72	72	48	48	HGG	T
**Pt21**	18	12	18	48	36	36	72	72	72	48	48	LGG	F-T
**Pt22**	18	12	18	48	36	36	72	72	72	48	48	LGG	T-P
**Pt23**	18	12	18	48	36	36	72	68	72	48	48	HGG	P
**Pt24**	18	12	18	48	36	36	72	72	72	48	48	HGG	T-P
**Pt25**	18	12	18	48	36	36	72	72	72	48	48	LGG	F
**Pt26**	18	12	18	48	36	36	72	72	72	48	48	HGG	T
**Pt27**	18	12	18	48	36	36	72	72	72	48	48	LGG	F-T
**Pt28**	18	12	18	48	36	36	72	72	72	48	48	HGG	T
**Pt29**	18	12	18	48	36	36	72	72	72	48	48	LGG	P
**Pt30**	18	12	18	48	36	36	72	72	72	48	48	LGG	T
**Pt31**	18	12	18	48	36	36	72	72	72	48	48	LGG	P
**Pt32**	18	12	18	48	36	36	72	72	72	48	48	LGG	F
**Pt33**	18	12	18	48	36	36	72	72	72	48	48	HGG	T
**Pt34**	18	12	18	48	36	36	72	69	72	48	48	HGG	P
**Pt35**	18	12	18	48	36	36	72	72	72	45	48	LGG	T
**Pt36**	18	12	18	48	36	36	72	72	72	47	48	HGG	P
**Pt37**	18	12	18	48	36	36	72	72	72	48	48	LGG	P
**Pt38**	18	12	18	48	36	36	72	72	72	48	48	LGG	F
**Pt39**	18	12	18	48	36	36	72	72	72	47	48	HGG	T
**Pt40**	18	12	18	48	35	35	70	72	72	48	48	HGG	T
**Pt41**	18	12	18	48	35	35	70	68	69	48	48	other	F

Supplementary Table 1 Scores and Clinical Data

**Pt42**	18	12	18	48	36	34	70	68	72	48	48	LGG	F
**Pt43**	18	12	18	48	36	34	70	72	72	48	48	HGG	F
**Pt44**	18	12	18	48	35	35	70	72	72	48	48	LGG	F-T
**Pt45**	18	12	18	48	35	35	70	72	72	48	48	HGG	T
**Pt46**	18	12	18	48	35	35	70	72	72	48	48	HGG	F-T
**Pt47**	18	12	18	48	35	35	70	72	72	48	48	other	P
**Pt48**	18	12	18	48	35	35	70	70	72	48	48	LGG	F
**Pt49**	18	12	18	48	36	33	69	72	68	48	48	LGG	T
**Pt50**	18	12	18	48	35	34	69	70	72	48	48	HGG	F
**Pt51**	18	12	18	48	36	31	67	67	72	48	48	HGG	T
**Pt52**	18	12	18	48	32	35	67	69	72	48	48	LGG	T-P
**Pt53**	18	12	18	48	36	30	66	70	70	48	48	HGG	F-T
**Pt54**	18	12	18	48	36	30	66	72	72	48	48	LGG	P
**Pt55**	18	12	18	48	30	36	66	66	70	48	48	HGG	T
**Pt56**	18	12	18	48	30	31	61	64	69	48	48	HGG	F
**Pt57**	18	12	18	48	27	26	53	72	72	48	48	HGG	F
**Pt58**	18	12	18	48	25	26	51	72	72	48	48	HGG	T-P
**Pt59**	18	12	18	48	32	21	50	55	72	48	48	HGG	P
**Pt60**	18	12	18	48	16	14	30	67	72	48	48	HGG	P
**Pt61**	18	12	17	47	35	35	70	72	72	48	48	HGG	F-T
**Pt62**	18	12	17	47	36	33	69	72	72	48	48	HGG	F-T
**Pt63**	18	12	17	47	28	16	44	44	62	48	48	HGG	P
**Pt64**	18	12	17	47	20	23	43	72	72	48	48	HGG	P

Supplementary Table 1 Scores and Clinical Data

**Pt65**	18	10	18	46	17	13	30	60	72	48	48	HGG	P
**Pt66**	18	12	15	45	36	36	72	72	72	48	48	HGG	F
**Pt67**	18	12	15	45	36	34	70	70	72	45	48	HGG	F
**Pt68**	18	12	15	45	35	27	62	72	61	48	48	HGG	T-P
**Pt69**	18	12	14	44	36	32	68	72	72	48	48	LGG	T-P
**Pt70**	18	11	13	42	36	36	72	72	72	47	48	HGG	P
**Pt71**	18	9	13	40	30	32	62	60	72	34	48	LGG	F
**Pt72**	17	12	10	39	28	27	55	68	72	48	48	HGG	P
**Pt73**	18	12	8	38	21	18	39	72	59	48	48	HGG	P
**Pt74**	16	10	9	35	35	35	70	70	68	48	48	HGG	P
**Pt75**	15	8	12	35	30	18	48	72	72	48	48	LGG	P
**Pt76**	18	8	7	33	30	18	48	67	72	48	48	HGG	P
**Pt77**	16	6	6	28	16	17	33	65	72	48	48	HGG	P
**Pt78**	11	8	7	26	24	17	41	72	70	48	48	LGG	F
**Pt79**	10	4	6	20	31	31	62	68	70	41	48	HGG	T-P

Legend
Pt = Patient
HGG = high grade glioma
LGG = low grade glioma
Other = meningioma
P = Parietal
F = Frontal
T = Temporal
T-P = Temporo-Parietal
F-T = Fronto-Temporal
